# Diagnosis model for assessing chronic thromboembolic pulmonary hypertension in high-altitude pulmonary embolism patients: a machine learning approach

**DOI:** 10.3389/fmed.2025.1666574

**Published:** 2025-10-07

**Authors:** Entong Fan, Jiangping Ma, Yanjun Zhang, Boning Yang, Gulinigeer Zhakeer, Yini Huang, Qing Yu, Yanxi Zeng, Ma Mi

**Affiliations:** ^1^State Key Laboratory of Cardiovascular Diseases and Medical Innovation Center, Shanghai East Hospital, School of Medicine, Tongji University, Shanghai, China; ^2^Department of Neurology, Tongren Hospital, Shanghai Jiao Tong University School of Medicine, Shanghai, China; ^3^Department of Cardiology, Shigatse People’s Hospital, Shigatse, Tibet, China; ^4^Department of Cardiology, Shanghai Tenth People’s Hospital, School of Medicine, Tongji University, Shanghai, China; ^5^Tongji University School of Medicine, Shanghai, China

**Keywords:** predictive learning models, machine learning, pulmonary embolism, pulmonary disease, chronic obstructive

## Abstract

**Background:**

Patients with pulmonary embolism (PE) at high altitude face an increased risk of developing chronic thromboembolic pulmonary hypertension (CTEPH). This study aims to establish a diagnosis model of CTEPH patients at high altitude to optimize early screening.

**Methods:**

A retrospective cohort of CTEPH and PE patients was rigorously selected through inclusion/exclusion criteria. Clinical data encompassing biochemical profiles, echocardiography, and CT angiography (CTA) were collected, yielding 103 candidate variables. Feature parameters were screened using the Boruta algorithm, followed by predictive model development with seven machine learning architectures. The optimal model was identified based on area under the curve (AUC). The optimal Random Forest model was subsequently interpreted through Shapley Additive Explanations (SHAP) to quantify feature contributions.

**Results:**

Among 57 PE patients, 44% met echocardiographic criteria for pulmonary hypertension following PE. Diameter of right atrium, diameter of right ventricle, Vessel-Grade (of embolization) and Sup-inferior (superior or inferior of embolization) were key identified predictors. Random Forests model had the highest AUC of 0.842. Enlarged right heart, embolization of small vessels and superior pulmonary artery embolism increased the risk of CTEPH, while normal right heart structure and isolated inferior pulmonary embolism reduced it.

**Conclusion:**

The Random Forests model demonstrated potential for detecting CTEPH in PE patients, enabling early and rapid pulmonary hypertension assessment.

## 1 Introduction

Pulmonary hypertension (PH) is a complex pathophysiological disorder marked by respiratory and circulatory symptoms (1), with delayed diagnosis and poor prognosis often leading to right heart failure (2). Five groups of PH are recognized, all defined by a mean pulmonary artery pressure (mPAP) > 20 mmHg as measured by right heart catheterization (RHC) (3). Among these, chronic thromboembolic pulmonary hypertension (CTEPH), classified as group 4 PH, may develop in a subset of patients after pulmonary embolism (PE), with reported incidence rates of 2%–3% (95% CI 1.5–4.4) in low-altitude populations based on studies encompassing over 2000 patients (4). In remote high-altitude regions, healthcare resource limitations including restricted access to right heart catheterization (RHC) complicate early CTEPH detection (5).

Chronic thromboembolic pulmonary hypertension is characterized by the organization/fibrosis of thrombi obstructing the proximal pulmonary arteries, accompanied by distal microvasculopathy, endothelial dysfunction, and inflammatory responses (6). Critically, the hypoxic environment at high altitude is not merely a logistical challenge but a central physiological driver of disease. Chronic hypoxia can induce pulmonary vasoconstriction and vascular remodeling, amplifying the pulmonary vascular resistance caused by thromboembolic obstructions and leading to a more severe phenotype of CTEPH that is distinct from low-altitude populations (7). However, current research on CTEPH in high-altitude regions remains limited, and findings from low-altitude studies may not be generalizable to populations with unique physiological adaptations (8). The physiological state of high-altitude (>2500 m) residents is different from low-altitude (<1000 m) residents. Long-term hypoxia may lead to progressive chronic mountain sickness (CMS) characterized by severe symptomatic excessive erythrocytosis and hypoxia-induced PH (9, 10). Hypoxic pulmonary vasoconstriction is commonly thought to underlie severe PH at high altitude (11, 12). However, long-term exposure to hypoxia at high altitude is the risk factor for both PE and hypoxia-induced PH (5, 13), thus most CTEPH patients at high altitude may actually have multifactorial PH caused by both chronic PE and hypoxia (14). This complexity may result in distinct imaging features compared to low-altitude areas, complicating diagnosis. Investigating imaging characteristics of high-altitude CTEPH is essential to the risk assessment of PE patients developing to CTEPH.

Current studies on CTEPH prediction models show a trend toward integrating diverse methods and parameters, yet face limitations in standardization, large-scale validation, clinical consensus, and applicability only to plain patients (15, 16). Risk prediction models utilize mathematical equations to estimate the likelihood of the individual contracting a disease or experiencing specific outcomes in the future (17). This superiority stems from their unparalleled flexibility in capturing non-linear variable relationships and detailed data patterns, leading to more precise predictions and enhanced model performance (18). Moreover, ML techniques are more robust to outliers and less sensitive to extreme values than logistic regression, providing superior handling of complex clinical data (19, 20). Despite the increasing use of ML in healthcare, its application among high-altitude populations remains scarce, and no prediction models exist for CTEPH. This study aims to explore the imaging characteristics and establish a ML-based prediction model to improve the early diagnosis and management of CTEPH in high-altitude areas.

## 2 Materials and methods

### 2.1 Study design and population

Blood samples were collected at 7 a.m. in the fasting state. The inclusion criteria were as follow: (1) male or female between the age of 18 and 85; (2) admission to Shigatse People’s Hospital between August 2022 and August 2024; (3) a documented history of residing in high-altitude areas for more than 20 years; (4) initial diagnosis suggesting PH; (5) echocardiographic pulmonary arterial systolic pressure (PASP) more than 50 mmHg; (6) CTA findings indicative of chronic thromboembolic disease, including vascular webs/bands, intimal irregularities, and abrupt vascular narrowing. The exclusion criteria for the retrospective training set included: (1) individuals with contraindications for undergoing CTA; (2) a documented history of left heart disease or echocardiography signs thereof, which includes diastolic dysfunction, systolic dysfunction and valvular diseases; (3) excessive erythrocytosis (defined as Hb ≥ 19 g/dl for females and ≥21 g/dl for males); (4) the presence of other lung disease, severe hepatic or renal insufficiency; (5) pregnancy; (6) incomplete echocardiographic data; (7) PH associated with autoimmune diseases (for a detailed process of patient selection, please refer to Supplementary Figure 1). Moreover, CTEPH diagnosis required: (1) ≥3 months anticoagulation; (2) persistent PASP > 50 mmHg; (3) chronic embolism signs on CTA.

### 2.2 Echocardiography

According to the guideline from the American Society of Echocardiography (21), the transthoracic echocardiography was performed to measure the following indicators: the vertical and transverse diameter of the four chambers, main pulmonary artery diameter, peak tricuspid regurgitation velocity and the left ventricle ejection fraction (LVEF) (21). It is worth mentioning that the parameters of right atrium and ventricle were obtained using the right ventricle–focused apical four-chamber view (A4C), which optimizes right ventricular visualization by adjusting the transducer on the basis of the A4C. The main pulmonary artery diameter was measured in the pulmonic valve (PV)-focused parasternal short-axis (PSAX) view (21). The pressure gradient between the right atrium and ventricle was estimated using the modified Bernoulli equation, based on the tricuspid regurgitation pressure gradient (TRPG) (22). Right atrial pressure was assessed and further combined with TRPG to calculate PASP (23).

### 2.3 Computed tomography angiography (CTA) acquisition

Computed tomography angiography scans were performed from the lung apex to the diaphragm using United Imaging scanners with patients in the supine position during an inspiratory breath-hold. Scanning parameters were as followed: tube voltage of 120 kVp, tube current 300 mA, tube rotation time 0.3 s, collimator width 64 mm × 0.625 mm. Iodinated contrast agent (Lomeprol, Bracco Sine, China, 400 mgL/ml) was injected through the median cubital vein with double-syringe power injector. The intelligent tracking mode was used, with 60 ml of contrast agent injected at 4 ml/s. Scanning was initiated when the density of the main pulmonary artery reached the preset threshold of 80 HU (24). CTA measurements included: Atrial diameter, bronchus and arterial diameter (assessed in the four-chambered transversal view), artery-bronchus ratio (ABR) (measured and calculated at the junction of the main bronchus).

### 2.4 Data preprocessing and feature filtering

The initial dataset comprised 103 clinical and imaging variables. Feature selection was executed via the Boruta algorithm, which identifies statistically significant predictors by comparing each feature’s *Z*-value against that of the “shadow features.” Bootstrap resampling was subsequently applied for model validation to mitigate overfitting concerns (25). In each iteration, the algorithm duplicates and shuffles all actual features to create “shadow features.” Then, a random forest model is used to obtain the *Z*-value for each attribute. The *Z*-value of the shadow is created by random shuffling of the actual features. A real feature was deemed significant if its Z-score consistently exceeded the maximum Z-score of shadow features across multiple independent trials. This process ensured robustness against random correlations and overfitting.

### 2.5 Model development and comparison

After feature selection, seven ML algorithms were tested for model construction: Logistic Regression (LR), K-Nearest Neighbors (KNN), Support Vector Machine (SVM), Decision Tree (DT), Random Forest (RF), Extreme Gradient Boosting (XGBoost), and Multilayer Perceptron (MLP). Due to the limited sample size, the full dataset was used for model development. The bootstrap method was employed for the purpose of validating predictive models (26). The Bootstrap method, a highly efficacious non-parametric statistical approach, estimates population characteristics through iterative sample resampling without requiring other assumptions or the addition of new samples. It circumvents cross-validation-induced sample attrition while generating stochastic datasets for internal model validation, using the following parameters as evaluation tools: area under the curve (AUC), accuracy, and brier score. Subsequently, the accuracy of model predictions was evaluated through the utilization of calibration curves, while the clinical applicability of the model was appraised through the implementation of a decision curve analysis. Potential Boruta-RF interdependence was mitigated by: (1) External validation using SHAP; (2) Comparing feature rankings with alternative algorithms.

### 2.6 Model explanation

Shapley Additive Explanations (SHAP) was used to provide both local and global model interpretability (27). SHAP is a game theory-based approach that assigns Shapley values to quantify each feature’s contribution to model predictions. SHAP force plots provided an intuitive visualization of how different features affect an individual prediction. SHAP for global interpretation not only revealed about the importance of features but also their relationship with the output. In our work, SHAP feature importance assessment was used for global interpretation of the developed baseline model. To identify the main predictors of CTEPH in PE patients in the high-altitude, we calculated the importance of ranking features in the final model. SHAP also provided examples of how individual predictions can be explained locally.

### 2.7 Statistical analysis

The Shapiro-Wilk test was used to assess the normality of variables. Normally distributed data, non-normal distribution data and categorical data were presented as mean ± standard deviation (SD), median [interquartile range (IQR)] and frequency, respectively. The dataset contained no missing values, and outliers identified via the interquartile range (IQR) method were individually assessed. Only those attributable to measurement error were removed, while others were retained and analyzed using robust statistical methods. Two-tailed unpaired *t*-test and Mann-Whitney U test were used for quantitative data analysis of differences between groups as appropriate. Qualitative data were analyzed using the chi-square test. Receiver operating characteristic (ROC) curve was constructed and the area under the ROC curve (AUC) was calculated to assess the predictive performances of the scoring models. Optimal cut-off values were determined by maximizing the Youden index: sensitivity + specificity − 1. Statistical analyses were conducted using SPSS 25.0 (version 25.0; IBM Corp., Armonk, NY, USA), R 4.4.1, and Python 3.12. The 2-sided *P* < 0.05 was considered statistically significant.

## 3 Results

### 3.1 Baseline characteristics of high-altitude CTEPH and PE patients

We collected data from PE patients at high altitude and divided them into two groups: the simple PE group and the CTEPH group, based on echocardiographic PASP (>50 mmHg or ≤50 mmHg) (28). The cohort consisted of 57 subjects (average age 63 years, 47% males), with 32 patients in the PE group and 25 patients in the CTEPH group (Supplementary Table 1). The two groups exhibited no significant differences in terms of age, sex and other baseline clinical characteristics, including D-dimer, brain natriuretic peptide (BNP), and C-reactive protein (CRP). In summary, these baseline characteristics reflect disease presentation adaptations unique to high altitude. This understanding ultimately enhances the interpretation of our results and improves the accuracy of CTEPH risk assessment in this specific environment.

### 3.2 Echocardiography characteristics

Echocardiography is the most commonly used non-invasive evaluation method for evaluating patients with PH. In this study, echocardiographic parameters related to cardiac structure and hemodynamics were analyzed (Table 1). The CTEPH cohort demonstrated significant right heart remodeling, manifested by: (1) right ventricle and atrium (RV/RA) enlargement in transverse and vertical diameters (RV, *P* < 0.05; RA, *P* < 0.05); (2) main pulmonary artery dilation (MPAD, *P* = 0.012); (3) elevated TRPG and PASP (*P* < 0.001). Consequently, CTEPH patients demonstrated significantly higher prevalence of right heart dysfunction (*P* < 0.001), defined as ≥2 criteria from: (1) RA/RV dimensional enlargement; (2) MPAD dilation. Conversely, no left heart dimensional (LA/LV) or functional (LVEF) differences were observed versus controls.

**TABLE 1:** Comparisons of echocardiographic parameters.

Characteristic		Total (*N* = 57)	PE (*N* = 32)	CTEPH (*N* = 25)	*P*-value
RAD1, mm	Enlarge (%)	25 (44)	6 (19)	19 (76)	0.000^a^
RAD2, mm	Enlarge (%)	36 (63)	15 (47)	21 (84)	0.004^a^
RVD1, mm	Enlarge (%)	19 (33)	4 (13)	15 (60)	0.000^a^
RVD2, mm	Enlarge (%)	32 (56)	14 (44)	18 (72)	0.033^a^
LAD1, mm	Enlarge (%)	22 (39)	10 (31)	12 (48)	0.197
LAD2, mm	Enlarge (%)	14 (25)	6 (19)	8 (32)	0.249
LVEDD, mm	Enlarge (%)	12 (21)	7 (22)	5 (20)	0.863
LVESD, mm	Enlarge (%)	14 (25)	7 (22)	7 (28)	0.594
MPAD, mm	Enlarge (%)	28 (49)	11 (34)	17 (68)	0.012^a^
Sign of right heart dysfunction	0 or 1 (%)	21 (37)	17 (53)	4 (16)	0.000^a^
	≥2 (%)	36 (63)	15 (47)	21 (84)	
TRPG, mmHg		38 (29, 50)	25 (24, 32)	54 (47, 59)	0.000^a^
PASP, mmHg		48 (34, 62)	33 (31, 36)	67 (60, 70)	0.000^a^
LVEF, %		58 (50, 65)	59 (52, 66)	57 (47, 67)	0.273

Data are median (P_25_, P_75_), the categorical variables are presented as absolute numbers (percentages). Group differences were assessed by chi-square test or Kruskal-Wallis H tests. LAD1, transverse diameter of left atrium; LAD2, vertical diameter of left atrium; LVEDD, left ventricle end-diastolic diameter; LVEF, left ventricular ejection fraction. LVESD, left ventricle end-systolic diameter; MPAD, main pulmonary artery diameter measured by echocardiography; PASP, echocardiographic pulmonary arterial systolic pressure estimate; RAD1, transverse diameter of right atrium; RAD2, vertical diameter of right atrium; RVD1, transverse diameter of right ventricle; RVD2, vertical diameter of right ventricle; TRPG, tricuspid regurgitation differential pressure.

*^a^P* < 0.05: the group difference assessed by the Mann-Whitney U test or chi-square test was significant. CTEPH diagnosis based on echocardiographic PASP > 50 mmHg without RHC confirmation.

### 3.3 CTA characteristics

**FIGURE 1:**
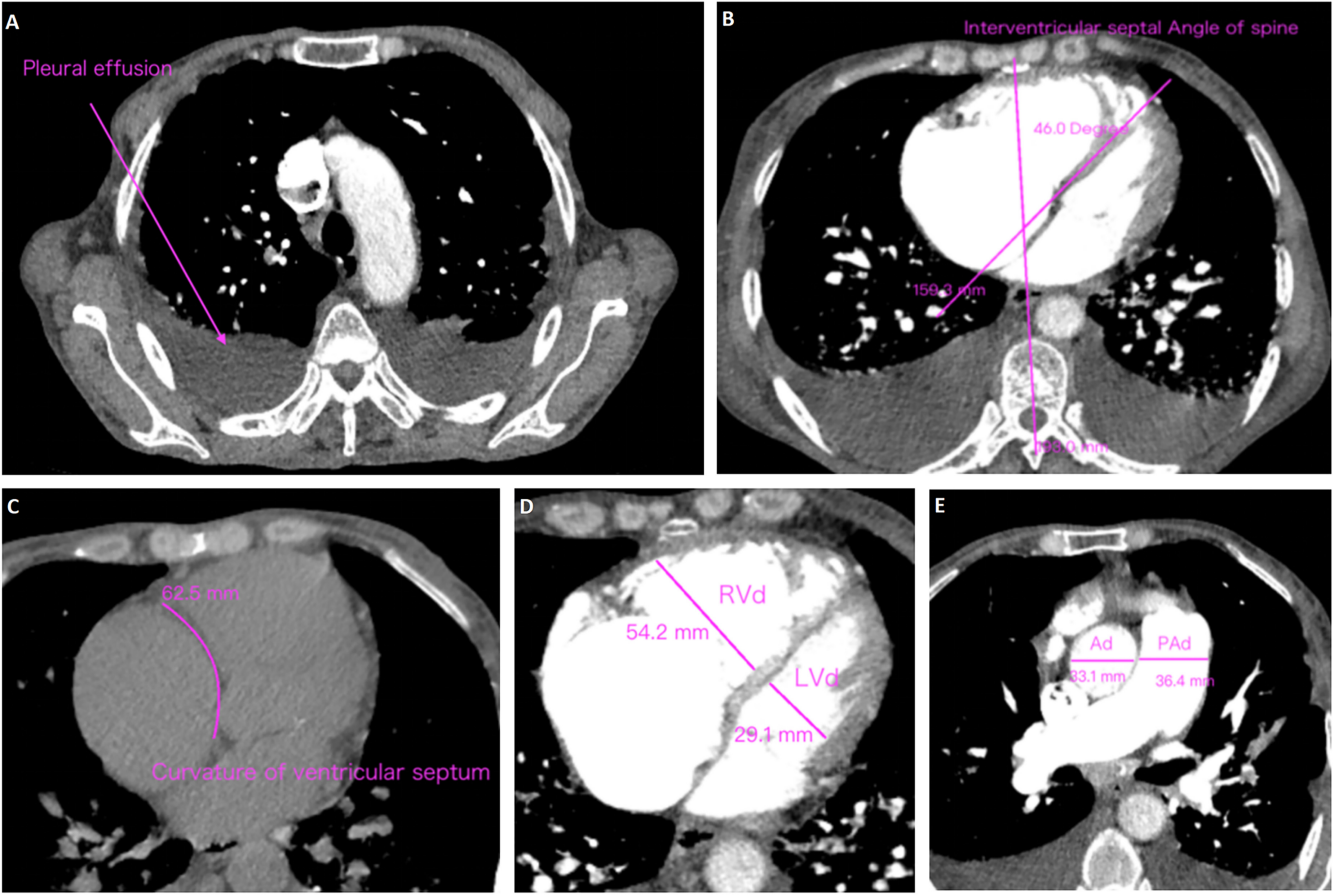
Methods of measurement and judgment of CTA signs. **(A)** Pleural effusion; **(B)** curvature of ventricular septum; **(C)** interventricular septal angle of spine; **(D)** right ventricular diameter (RVd)/left ventricular diameter (LVd); **(E)** pulmonary artery diameter (PAd)/aorta diameter (Ad).

CT angiography is increasingly recognized as a valuable diagnostic tool for CTEPH due to its ability to provide rapid, non-invasive visualization of the pulmonary vasculature and facilitate the evaluation of right heart load. Unlike echocardiography which primarily evaluates cardiac functional consequences, CTA directly delineates pulmonary arterial obstructions, thrombus morphology, and small-vessel pathology, thereby enhancing diagnostic accuracy. Accordingly, pulmonary CTA data were collected for all subjects to comprehensively assess the radiologic characteristics of CTEPH in high-altitude populations (Table 2 and Supplementary Table 2). The results showed that patients in CTEPH group were more likely to have enlarged left upper pulmonary bronchus diameter (*P* = 0.002), a reduced cardiothoracic ratio (*P* = 0.010), and a thinner interventricular septum (IVS) (*P* = 0.038). Additionally, we analyzed pleural effusion, ventricular septum curvature, and spinal cord interventricular septum angle in both the PE and CTEPH groups (Figure 1) (29). The results indicated no statistically significant differences in these parameters between the two groups. Importantly, pulmonary embolism in CTEPH patients were more likely to occur in tertiary pulmonary arteries (*P* = 0.008). This may be due to the smaller diameter of subsegmental arteries (diameter < 2 mm), making them more prone to complete thrombotic occlusion (30), or due to impaired right heart venous return propelling thrombi toward distal vessels (31).

**TABLE 2:** Comparisons of CTA parameters.

Characteristic	Total (*N* = 57)	PE (*N* = 32)	CTEPH (*N* = 25)	*P*-value
Pulmonary embolism location				
Left (%)	22 (39)	10 (31)	12 (48)	0.394
Upper (%)	15 (81)	3 (9)	12 (48)	0.662
Embolization vessel grade				
Grade 1 (%)	4 (7)	1 (3)	3 (12)	0.188
Grade 2 (%)	21 (37)	15 (47)	6 (24)	0.258
Grade 3 (%)	24 (42)	15 (47)	9 (36)	0.686
Grade 2 + 3 (%)	6 (11)	1 (3)	5 (20)	0.891
Grade 1 + 2 + 3 (%)	2 (4)	0	2 (8)	0.655
Primary vascular embolism (%)	53 (93)	31 (97)	22 (88)	0.197
Secondary vascular embolism	32 (56)	16 (50)	16 (64)	0.295
Tertiary vascular embolism	8 (14)	1 (3)	7 (28)	0.008^a^
Thrombus morphology				
Acute	30 (53)	16 (50)	14 (56)	1.000
Chronic	27 (47)	16 (50)	12 (48)	0.855
Left low ABR	1.6 (1.2, 1.9)	1.6 (1.2, 1.9)	1.5 (1.1, 1.9)	0.524
Left up ABR	1.6 (1.3, 1.8)	1.6 (1.4, 1.9)	1.6 (1.2, 1.8)	0.544
Right intermediate ABR	1.5 (1.2, 1.9)	1.6 (1.2, 1.9)	1.5 (1.2, 1.9)	0.611
Right up ABR	1.2 (0.9, 1.4)	1.1 (0.9, 1.4)	1.2 (0.9, 1.5)	0.365
Cardiothoracic ratio	0.6 (0.5, 0.7)	0.6 (0.5, 0.7)	0.6 (0.5, 0.6)	0.010^a^
IVS, mm	15.0 (10.0, 14.2)	16.6 (10.5, 15.2)	13.0 (8.9, 12.6)	0.038^a^
rRLA	1.6 (1.2, 1.8)	1.6 (1.2, 1.9)	1.7 (1.3, 1.9)	0.440
Pleural effusion, %	32 (55)	16 (50)	14 (56)	0.985
Curvature of ventricular septum	2 (3)	1 (3)	1 (4)	0.862
Spinal cord interventricular septum angle, degrees	42.4 ± 10.3	42.1 ± 11.1	42.9 ± 9.3	0.776

Data are mean ± SD or median (P_25_, P_75_), the categorical variables are presented as absolute numbers (percentages). ABR, pulmonary artery-bronchus ratio; IVS, interventricular septum thickness; rRLA, the ratio of right to left atrial diameter.

*^a^P* < 0.05: the group difference assessed by the Two-tailed unpaired *t*-test or Mann-Whitney U test was significant. CTEPH diagnosis based on echocardiographic PASP > 50 mmHg without RHC confirmation.

### 3.4 Selection of independent risk factors

**FIGURE 2:**
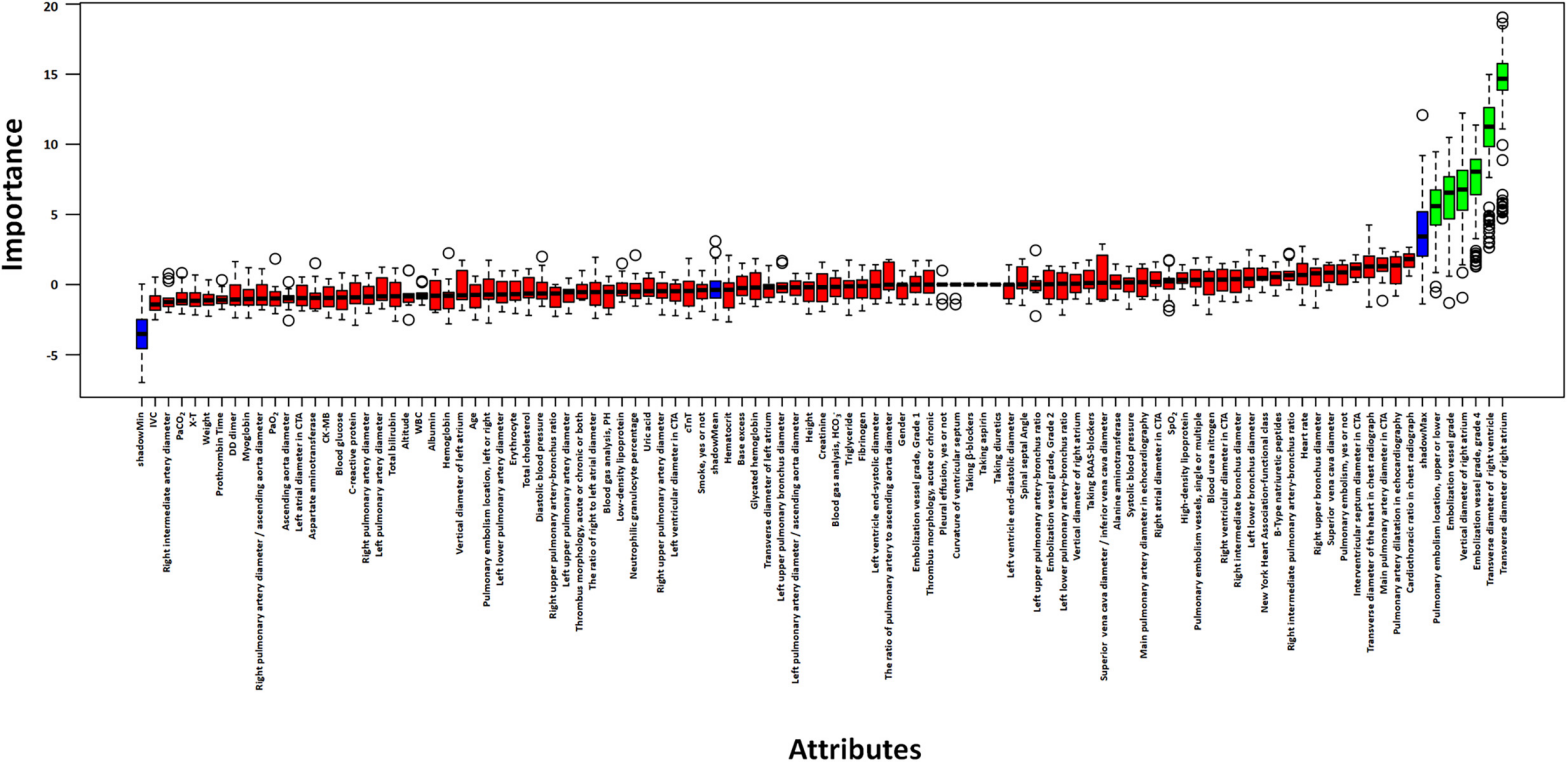
Selection of independent risk factors associated with CTEPH. Identify predictive factors for CTEPH using the Boruta algorithm.

Based on Boruta algorithm-derived feature importance rankings (Figure 2), we identified six critical predictors strongly associated with CTEPH development: (1) the location of blocked pulmonary vessels, (2) the grade of blocked pulmonary vessels, (3) whether the embolism is in a tertiary vessel, (4) right atrial transverse diameter (RAD1), (5) right atrial vertical diameter (RAD2), (6) right ventricular transverse diameter (RVD1). These findings align with the echocardiographic and CTA results, reinforcing that right heart structural changes and embolization characteristics are important predictive factors for CTEPH. Therefore, in high-altitude clinical practice, it is essential to closely monitor right heart function and the status of small-vessel embolization in patients with PE to aid in predicting the potential progression of CTEPH. Previous studies have investigated the location of pulmonary vascular obstructions and alterations in right heart structure and function. However, these factors have rarely been systematically analyzed as independent key predictors of CTEPH (32). Notably, the identification of embolism in tertiary pulmonary vessels and specific cardiac structural changes provides a novel perspective on CTEPH risk assessment. By integrating multiple pulmonary vascular and right heart-related parameters, this study enhances the predictive accuracy of CTEPH progression, ultimately improving early diagnosis and clinical decision-making.

### 3.5 Evaluation and comparison of the model

**FIGURE 3:**
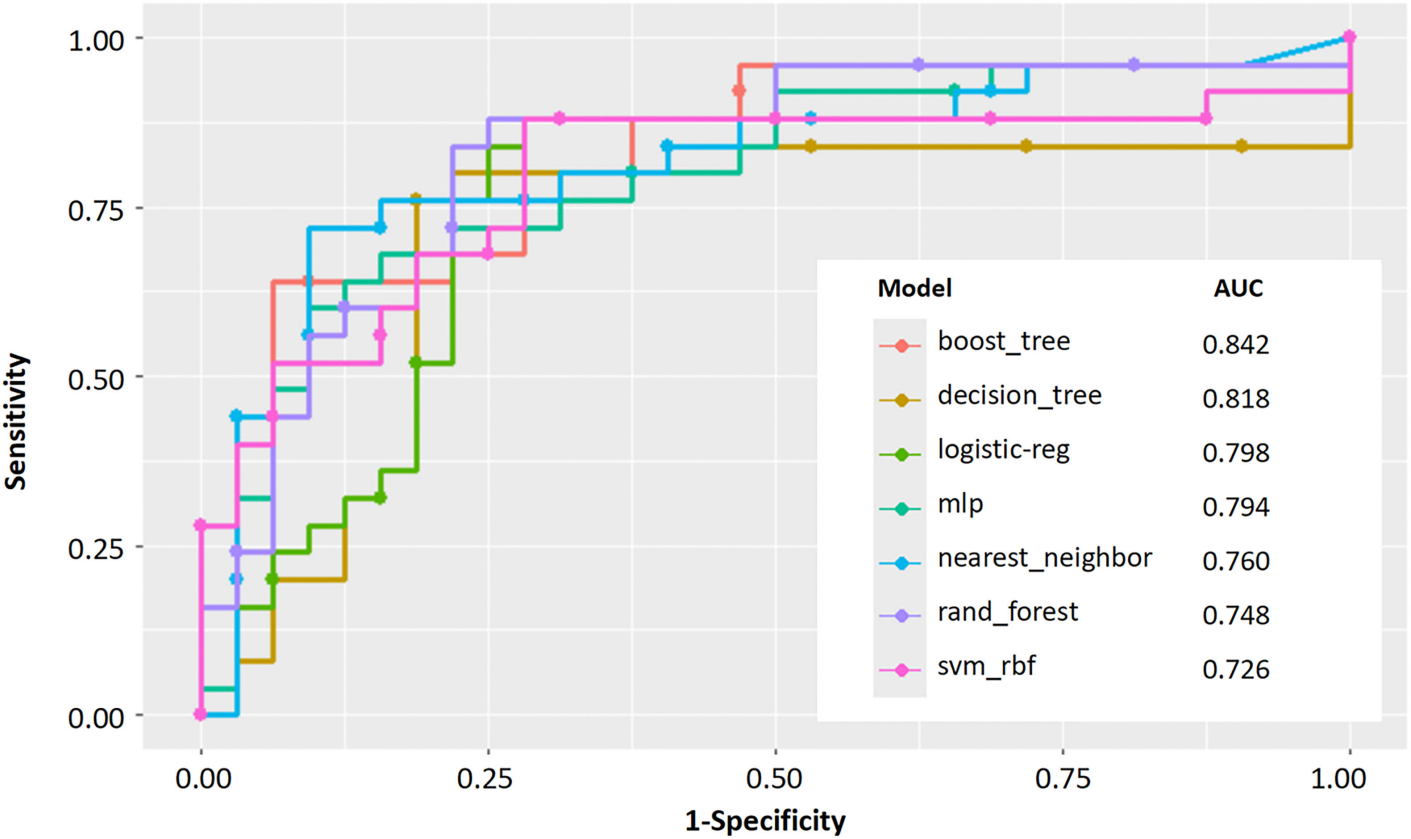
Performance evaluation of machine learning models for predicting. AUC curves for seven machine learning models.

The Random Forest model demonstrated superior predictive capability among seven evaluated algorithms, achieving a larger AUC (Figure 3). Consequently, we selected the Random Forest model for further analyses. Decision curve analysis (DCA) results indicated that utilizing the Random Forest model in our current study to predict CTEPH could provide greater clinical benefit within a specific threshold probability range (Supplementary Figures 2A, B). Moreover, the calibration curve demonstrated a significant agreement between predicted probabilities and actual outcomes, as shown in Supplementary Figure 2C. Overall, the Random Forest model showed robust predictive performance and aligned well with established medical risk factors for CTEPH, serving as a valuable decision-support tool. Ensuring clinicians understand why the model makes each prediction will foster trust and more effective use of the model, ultimately improving earlier and more accurate CTEPH detection.

### 3.6 Visualization of feature importance

**FIGURE 4:**
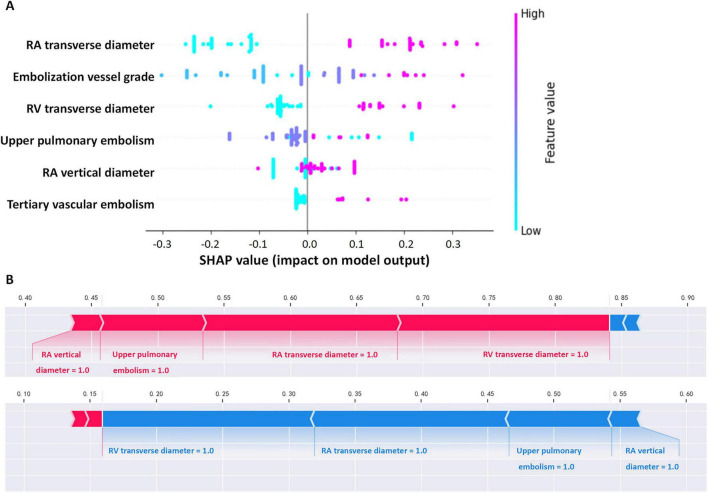
Interpretability of the metastasis model. **(A)** Contribution of each variable to the model. **(B)** Force plot for two outcomes for a single individual (in each graph, a red arrow denotes a positive effect on the outcome, while a blue arrow signifies a negative effect. The length of the arrow corresponds to the magnitude of the contribution. The output value represents the predicted probability of the outcome).

**FIGURE 5:**
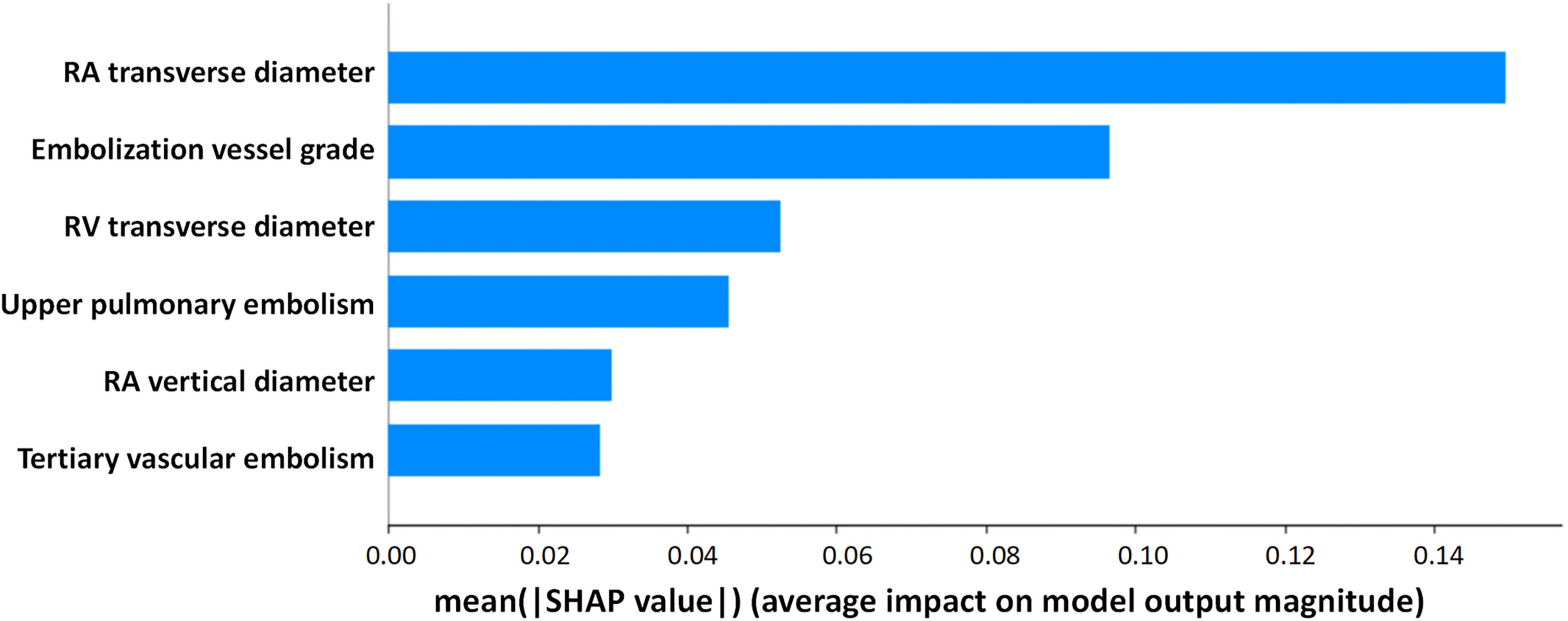
“SHAP” package to explain the importance of key variables to the model.

**FIGURE 6:**
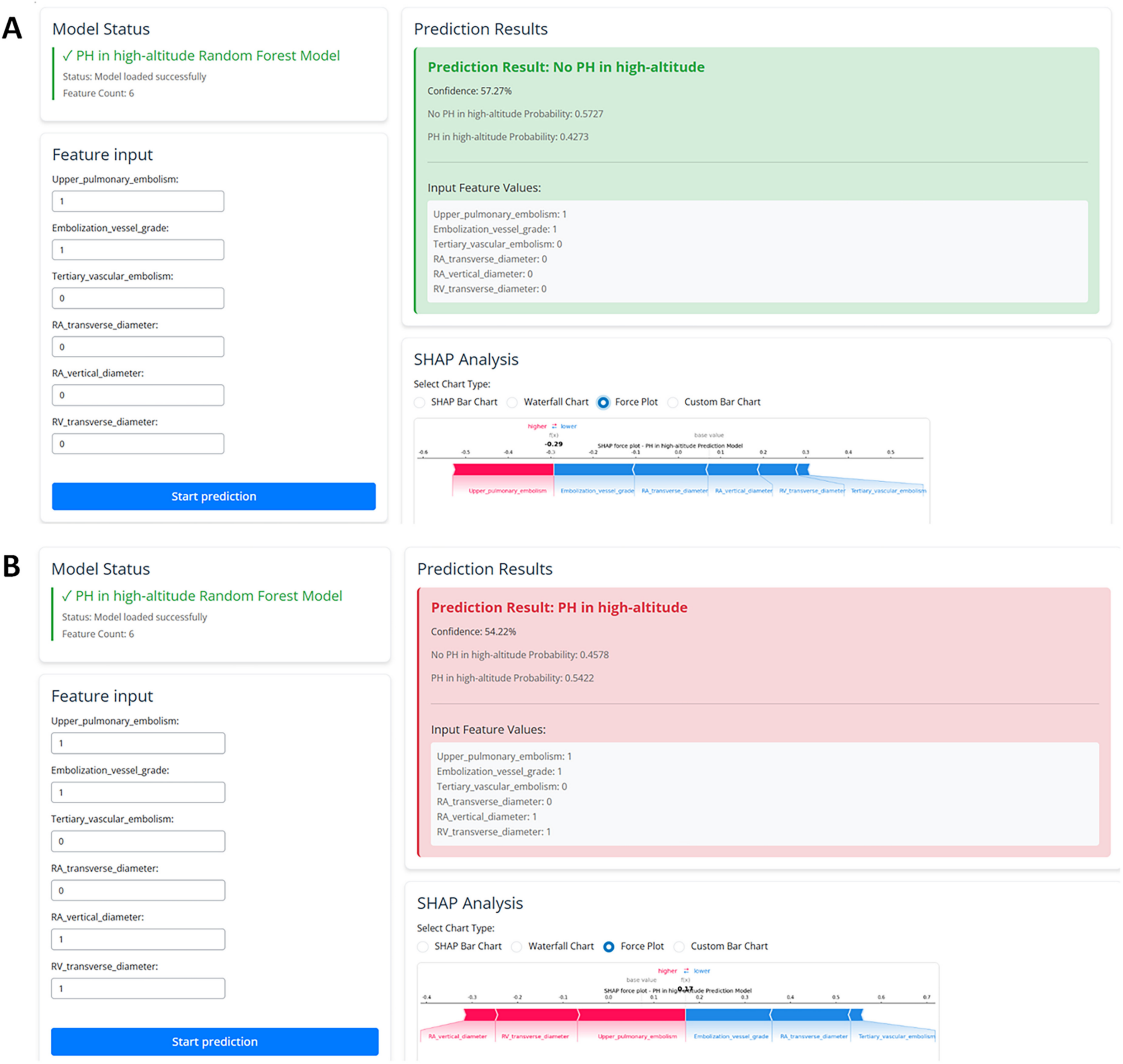
The web-based user-friendly prediction model, including prediction results with no PH **(A)** or PH in high-altitude **(B)**. (https://cq3qma-jiangping-ma.shinyapps.io/pahapp1/).

To enhance model interpretability, we used SHAP analysis to calculate the contribution of each variable in the Random Forest model. Figures 4, 5 illustrate the importance of the selected six predictive variables based on average absolute SHAP values. Figure 4B further depicts how these features influence the model’s predictions. Specifically, RAD2, location of blocked pulmonary vessels, RAD1, and RVD1 increased the risk of the prediction. Notably, RVD1 and RAD1 were associated with the highest predicted probabilities for CTEPH. In conclusion, CTEPH induces a series of structural and functional changes in the right heart, which can be effectively evaluated using various diagnostic methods. Additionally, studies suggested that the degree of right ventricular enlargement correlates closely with the severity of CTEPH (33, 34). Therefore, monitoring right heart enlargement in CTEPH patients at high-altitude holds significant clinical value for early diagnosis, treatment, and prognosis assessment. Finally, we have developed a more accessible and efficient web-based tool^1^ based on our optimal machine learning model, which can be deployed in other hospitals in high-altitude areas to enhance clinical applicability and societal benefit (Figure 6).

## 4 Discussion

**FIGURE 7:**
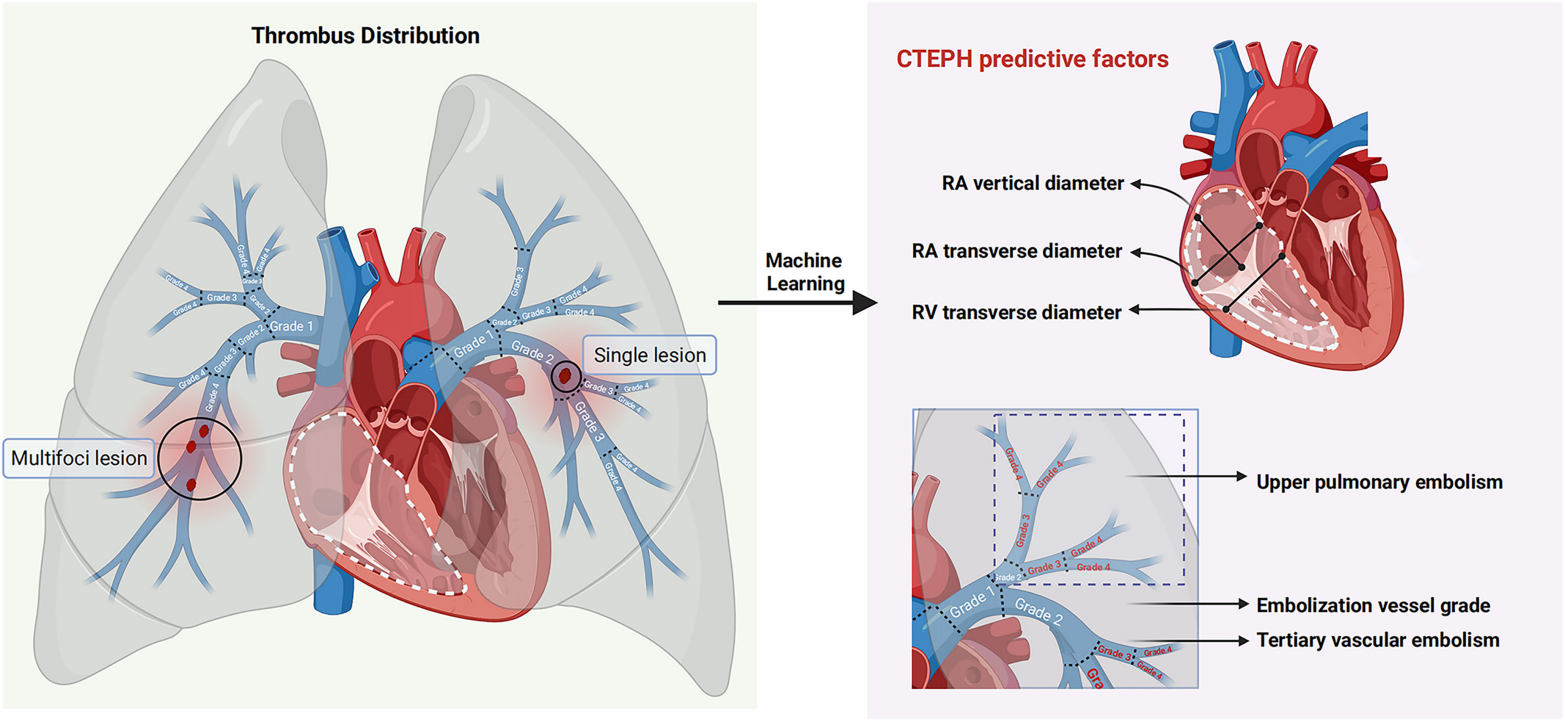
Predictors of CTEPH patients at high altitude. Through machine learning-driven feature selection, six predictive factors for CTEPH in patients with pulmonary embolism at high altitude were screened based on embolism location, thrombus property, thrombus morphology and cardiac structural changes.

This exploratory study provides preliminary insights into the risk factors of CTEPH in the high-altitude populations following a diagnosis of PE using ML techniques. We identified six diagnosis factors and successfully developed seven ML models for CTEPH identification, ensuring interpretability through SHAP. We determined that the Random Forest model showed promising discriminative ability and identified the grade of embolized vessels, the location of embolization, RAD1, RAD2, and RVD1 as the key factors for predicting CTEPH. This ML model enhances the ability to predict CTEPH in high-altitude PE patients, bridging a research gap and providing a novel tool for early diagnosis (Figure 7).

The present study built on previous prediction model using multiple linear regression and odds ratio (OR)-value assignments (35, 36). In recent years, with the change of prediction methods, the advanced ML classification algorithm has been found to improve the predictive accuracy and gradually provided valuable clinical decision-making support. In this study, ML techniques identified six risk factors that can predict the development of CTEPH in high-altitude PE patients, encompassing three major domains: embolization site, grade of embolized vessels, and structural changes of the right heart. Among the above risk factors, indicators related to right heart structure emerged as particularly significant. This finding aligns with research conducted in low-altitude populations, which suggested that right ventricular dilation and hypertrophy during the PE stage substantially increase the risk of CTEPH development, with mortality rates largely dependent on underlying comorbidities (37).

Although the identified risk factors share similarities with those in low-altitude populations, the impact of high-altitude environments cannot be overlooked. Long-term exposure to the hypoxic environment of the high-altitude induces pulmonary vasoconstriction, increasing pulmonary vascular resistance and pressure. High-altitude dwellers exhibit distinct pulmonary circulation differences compared to low-altitude populations. These include a reduced pulmonary circulation reserve capacity, sustained pulmonary artery contraction, and increased pulmonary vascular resistance (8, 38, 39). Furthermore, hypoxia may alter embolism distribution patterns, elevating small-vessel predictive value–a phenomenon requiring prospective validation.

Notably, previous studies have indicated elevated levels of D-dimer, BNP, and CRP in patients with CTEPH compared to PE patients in low-altitude area (40, 41). However, this differed from the conclusions of the present study, which might potentially be attributed to delayed medical consultation caused by limited healthcare resources in high-altitude regions, or the hypoxic environment at high altitude predisposing individuals to thromboembolic events, contributing to the development of high-altitude heart disease (38, 39). Right ventricular dysfunction, adverse remodeling, and associated hemodynamic abnormalities are key predictors of disease progression and mortality in CTEPH patients (42). Besides, our findings demonstrate that CTEPH is associated with more pronounced right heart dysfunction than simple PE, emphasizing that progressive right atrium and right ventricle dilation in CTEPH is a key pathophysiological feature underlying its clinical severity, as evidenced by right heart enlargement and hemodynamic alterations. Additionally, the results provide stronger clinical insights into the impact of CTEPH on cardiac structure and function at high-altitude.

Microvascular embolization holds particular importance in CTEPH, as it not only represents a core manifestation of distal microvasculopathy but also synergizes with chronic hypoxia to exacerbate pulmonary vascular remodeling. This process leads to more insidious and diffuse hemodynamic impairments, significantly affecting diagnostic challenges, treatment response, and long-term prognosis (43, 44). A particularly noteworthy finding of this study is that multiple lesions in more than grade 3 vessels were more likely to lead to CTEPH than large-vessel embolization. This may be attributed to the distinct hemodynamic changes in different grades of vascular branches (45). Previous studies have suggested that conditions such as low wall shear stress, low blood flow velocity, high blood viscosity coefficient, high whole - blood viscosity, and long cumulative residence time can all lead to thrombus formation (46, 47). At the same time, Ventilation - perfusion (V/Q) scanning has shown that abnormal pulmonary blood flow perfusion mainly occurs in proximal large vessels and distal small vessels. These vessels frequently exhibited intimal thickening, smooth muscle hyperplasia, luminal stenosis, and even occlusion, with microvascular lesions being particularly prevalent (48, 49). In addition, hypoxia was more likely to cause the contraction of small pulmonary vessels, triggering the body to produce a series of stress responses, leading to changes in blood flow status, damage to the vascular endothelium, and increased blood coagulation, thereby increasing the risk of thrombosis (50, 51). Taken together, these findings suggest that embolization of small pulmonary arteries at high altitude, combined with hypoxia-induced vascular remodeling, exacerbates the progression of pulmonary hypertension. Given that smaller-diameter vessels are more susceptible to complete occlusion, blood flow obstruction in the pulmonary circulation becomes more severe, leading to a greater likelihood of developing CTEPH.

This study offers innovative insights, yet it is important to recognize its limitations. Firstly, the sample size was relatively small, with only 57 patients meeting the stringent inclusion and exclusion criteria during the 2-years screening. Secondly, the absence of an independent validation cohort makes it difficult to comprehensively evaluate the generalization ability of the model. Additionally, hypoxia conditions at high altitude can also cause pulmonary hypertension. However, hypoxia-induced pulmonary hypertension (HAPH) patients were not included as a separate category for analysis, limiting the ability to fully evaluate the role of hypoxia in CTEPH development. To mitigate the confounding effects of hypoxia, variables were analyzed in different aspects before the process of model establishment. Statistical analysis and SHAP were used to compare the difference between patients of CTEPH and PE and the accuracy of the predicted model. As healthcare access improves in high-altitude regions, more CTEPH and PE patients will be included for model correction, and further studies with larger, multi-center cohorts and prospective designs are needed to validate our findings and refine the predictive model.

## 5 Conclusion

In this study, we found that PE patients at high altitude with multiple grade 3–4 small arterial emboli and upper pulmonary artery embolism are at a higher risk of developing CTEPH. Additionally, we constructed seven ML models and successfully created a stable ML model for identifying CTEPH in PE patients at high altitude, utilizing echocardiography and CTA data–both readily accessible and clinically applicable. The Random Forests model was the most efficient in detecting CTEPH, offering a reliable tool for clinical decision-making regarding diagnosis for CTEPH. Ultimately, the application of SHAP decision charts has facilitated the development of an early CTEPH identification framework. Besides, this model serves as a triage tool, not diagnostic; screen-positive patients require referral to RHC-equipped centers.

## Data Availability

The original contributions presented in this study are included in this article/Supplementary material, further inquiries can be directed to the corresponding authors.

## Footnotes

1
https://cq3qma-jiangping-ma.shinyapps.io/pahapp1/
