# Supplementary material for: Diagnosis model for assessing chronic thromboembolic pulmonary hypertension in high-altitude pulmonary embolism patients: a machine learning approach
Source: Front Med (Lausanne). 2025 Oct 7;12:1666574. doi: 10.3389/fmed.2025.1666574 (PMC12537356; doi:10.3389/fmed.2025.1666574)
Supplement: Supplementary file 1 [file Data_Sheet_1.docx]

Supplementary Material

# Supplementary Figures and Tables

## Supplementary Figures


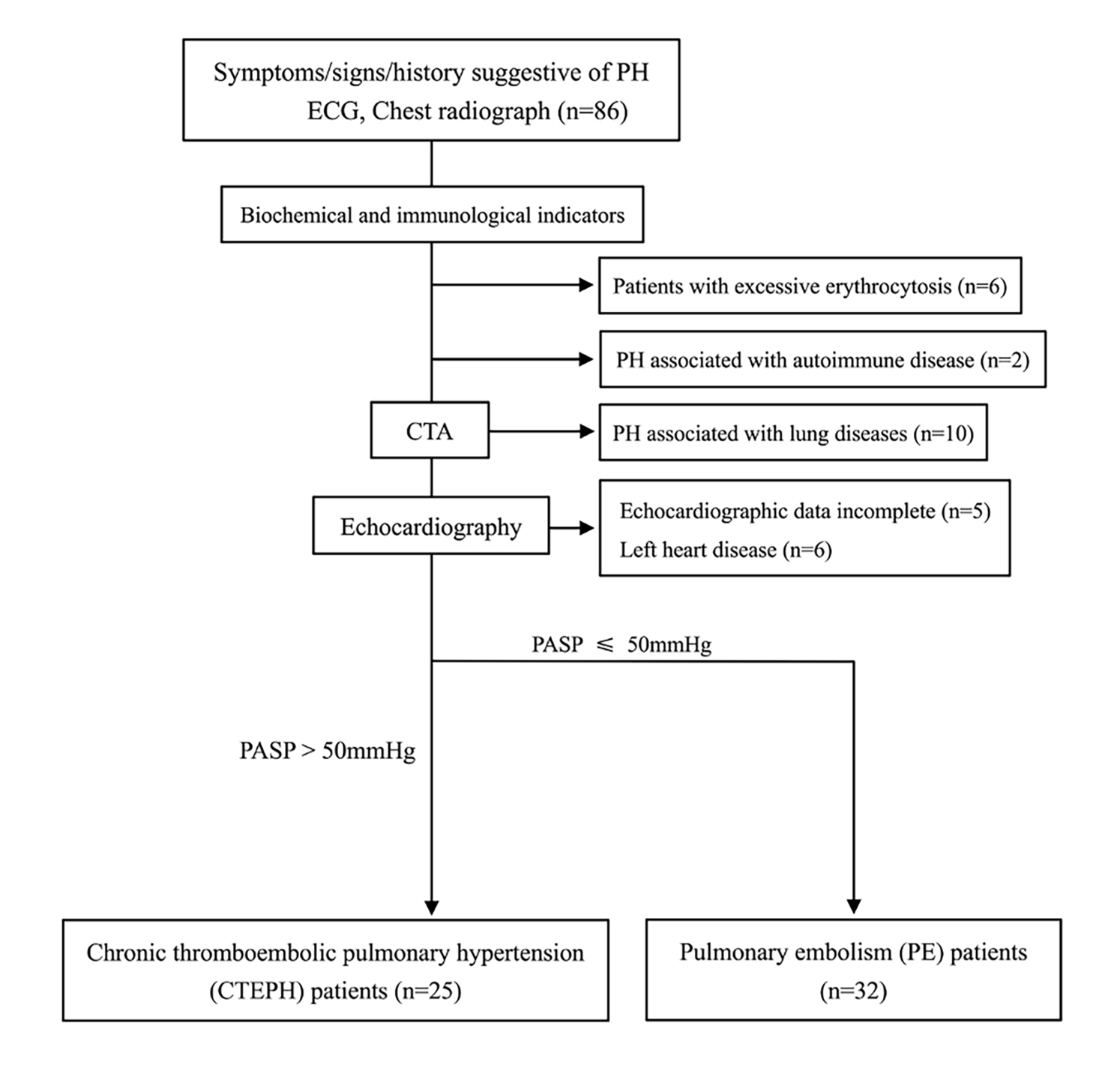


**Supplementary Figure 1 Flow chart of patients included and excluded.** The inclusion criteria were the following: 1) individuals of both male and female aged 18 to 85; 2) admission to Shigatse People’s Hospital between August 2020 and August 2022; 3) a documented history of residing in high-altitude areas for more than 20 years; 4) initial diagnosis suggesting PH; 5) performance of CTA during hospitalization. The exclusion criteria for the retrospective training set included: 1) excessive erythrocytosis (defined as Hb ≥ 19 g/dl for females and ≥ 21 g/dl for males); 2) PH patients associated with autoimmune diseases; 3) PH patients associated with lung diseases; 4) incomplete echocardiographic data; 5) have a documented history of left heart disease. Those with PASP > 50mmHg were classified as CTEPH patients, while those with PASP ≤ 50mmHg were classified as PE patients.


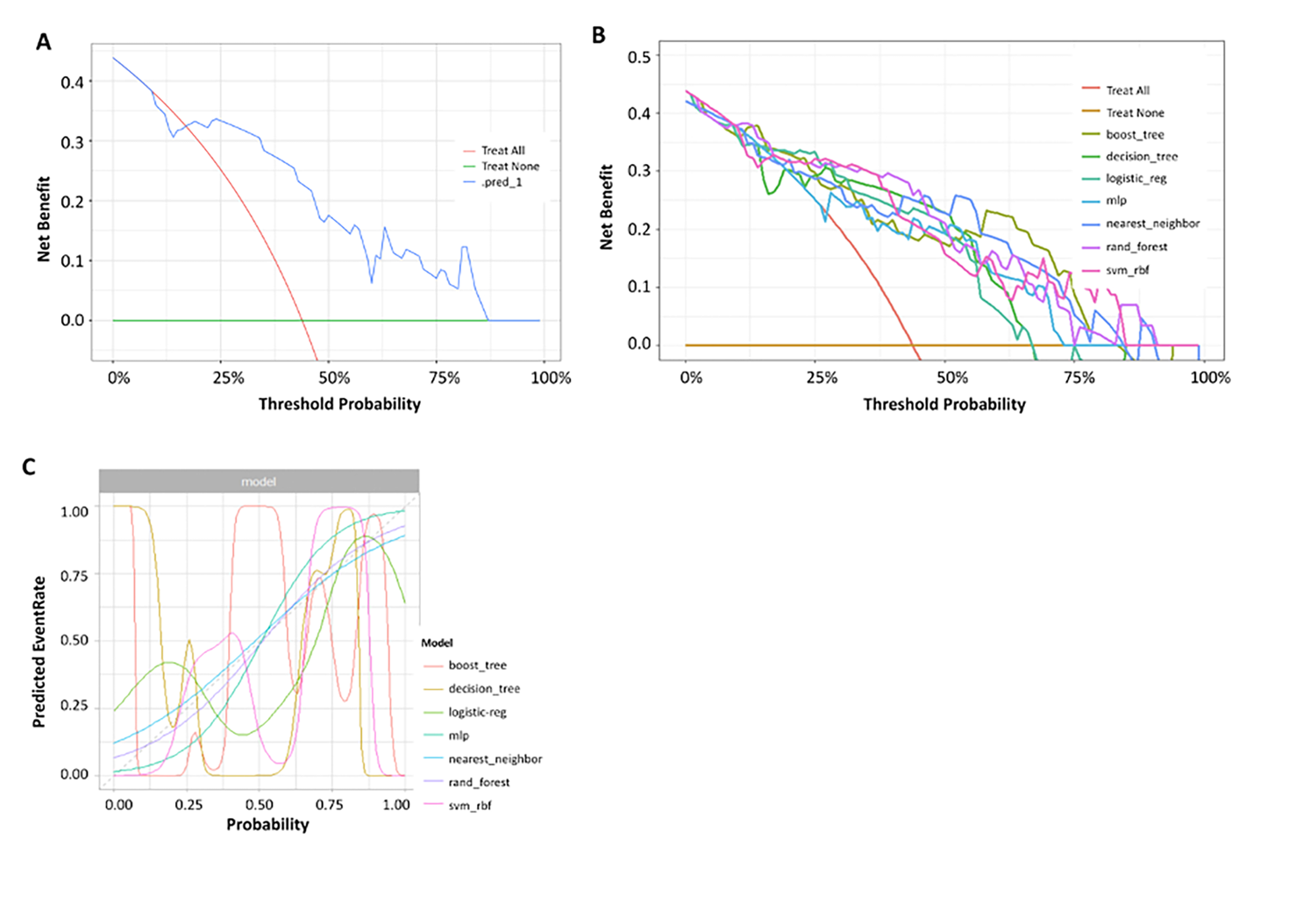


**Supplementary Figure 2 Performance evaluation of machine learning models for predicting.** A: DCA of the Random Forest model. Decision curve analysis: DCA; B: DCA of the seven prediction models; C: The calibration curve of the seven prediction models. The Random Forest model achieved a larger (better) AUC compared with the other models.

## Supplementary Tables

**Supplementary Table 1 Baseline characteristics of individuals**

| **Characteristic** | **Total**  **(N=57)** | **PE**  **(N=32)** | **CTEPH**  **(N=25)** | ***P* value** | |
| --- | --- | --- | --- | --- | --- |
| Gender Male (%) | 27 (47) | 17 (53) | 10 (40) | 0.329 | |
| Age, years | 63 (53, 75) | 63 (54, 75) | 64 (51, 74) | 0.872 | |
| Altitude, m | 4040 (3888, 4300) | 3984 (3822, 4300) | 4113 (3875, 4250) | 0.845 | |
| NYHA, FC |  |  |  | 0.121 | |
| Grade 1 (%) | 9 (16) | 5 (16) | 4 (16) |  |  |
| Grade 2 (%) | 7 (12) | 7 (22) | 0 (0) |  | |
| Grade 3 (%) | 35 (61) | 15 (47) | 20 (80) |  | |
| Grade 4 (%) | 3 (5) | 2 (6) | 1 (4) |  | |
| Smoking Yes (%) | 16 (28) | 10 (29) | 4 (16) | 0.549 | |
| HR, bpm | 89 (77, 98) | 93 (78, 100) | 85 (69, 98) | 0.253 | |
| SBP, mmHg | 130 (112, 144) | 128 (105, 137） | 132 (113, 148） | 0.687 | |
| DBP, mmHg | 84 ± 16 | 84 ± 15 | 84 ± 17 | 0.866 | |
| Weight, kg | 63 (55, 65) | 63 (56, 67） | 62 (54, 67） | 0.904 | |
| Height, cm | 162 (160, 168) | 163 (160, 168） | 162 (156, 167） | 0.349 | |
| BMI, kg/m^2^ | 23.69 (21.08, 25.39) | 23.55 (21.08, 24.62) | 23.88 (20.66, 27.19) | 0.676 | |
| BSA, m^2^ | 1.77 ± 0.15 | 1.78 ± 0.15 | 1.76 ± 0.16 | 0.489 | |
| VO_2_, ml/min | 275.51 ± 25.01 | 277.08 ± 24.60 | 273.52 ± 25.90 | 0.664 | |
| CRP, mg/l | 45.84 (3.01, 54.39) | 35.89 (5.28, 54.04) | 58.58 (2.14, 60.00) | 0.629 | |
| WBC, ×10^9^/l | 7.0 (4.4, 8.0) | 6.8 (4.3, 7.4) | 7.2 (4.4, 8.6) | 0.359 | |
| Neu, % | 68.1 (66.5, 81.2) | 70.6 (68.2, 80.8) | 64.9 (60.1, 82.8) | 0.629 | |
| Erythrocyte, ×10^12^/L | 5.18 (4.30, 6.24) | 5.13 (4.04, 6.29) | 5.24 (4.35, 6.16) | 0.981 | |
| HGB, g/l | 148 ± 42 | 151 ± 39 | 144 ± 46 | 0.652 | |
| Hematocrit, % | 47.1 ± 12.8 | 48.3 ± 13.8 | 45.7 ± 11.6 | 0.445 | |
| ALT, U/l | 65 (18, 60) | 48 (14, 59) | 87 (20, 72) | 0.346 | |
| AST, U/l | 73 (24, 65) | 54 (24, 65) | 99 (21, 68) | 0.987 | |
| Albumin, g/l | 36.4 (33.2, 40.0) | 36.7 (33.4, 42.2) | 36.2 (32.7, 39.0) | 0.891 | |
| Total bilirubin, μmol/L | 33.7 (17.7, 36.5) | 33.9 (19.7, 45.3) | 33.6 (14.2, 33.8) | 0.339 | |
| CREA, μmol/l | 91 (64, 94) | 96 (65, 94) | 85 (64, 96) | 0.699 | |
| UA, μmol/l | 388 (273, 485) | 376 (269, 470) | 403 (268, 531) | 0.635 | |
| BUN, mmol/l | 5.09 (2.97, 5.72) | 5.12 (3.01, 5.70) | 5.02 (1.86, 6.08) | 0.917 | |
| FIB, g/l | 3.76 (2.19, 3.99) | 3.92 (1.87, 4.44) | 3.55 (2.39, 3.90) | 0.682 | |
| D,D dimer, mg/l | 3.54 (1.36, 3.39) | 3.70 (1.36, 3.19) | 3.33 (1.00, 3.75) | 0.879 | |
| PT, s | 240.0 (12.3, 16.0) | 217.3 (12.1, 17.2) | 269.2 (12.7, 16.0) | 0.624 | |
| PH | 7.00 (7.11, 7.50) | 6.92 (7.00, 7.50) | 7.09 (7.34, 7.50) | 0.590 | |
| SaO_2_, % | 76 (73, 91) | 73 (72, 91) | 78 (75, 93) | 0.385 | |
| PaO_2_, mmHg | 48 (43, 54) | 47 (40, 53) | 49 (44, 59) | 0.479 | |
| PaCO_2_, mmHg | 34 (28, 38) | 34 (27, 38) | 34 (28, 40) | 0.658 | |
| BE, mmol/L | 2 (-3, 2) | 3 (-3, 2) | 0 (-5, 2) | 0.705 | |
| HCO_3_^-^, mmol/l | 25.7 (20.2, 26.4) | 28.3 (20.25, 26.35) | 22.4 (19.00, 25.89) | 0.469 | |
| TC, mmol/l | 2.95 (2.61, 3.21) | 2.90 (2.54, 3.16) | 3.02 (2.68, 3.40) | 0.296 | |
| TG, mmol/l | 0.97 (0.84, 1.04) | 1.00 (0.89, 1.05) | 0.92 (0.78, 1.02) | 0.139 | |
| HDL-C, mmol/l | 1.00 (0.81, 1.12) | 0.95 (0.64, 1.17) | 1.07 (0.95, 1.13) | 0.376 | |
| LDL-C, mmol/l | 1.77 (1.50, 2.00) | 1.73 (1.47, 1.94) | 1.82 (1.45, 2.09) | 0.658 | |
| CK-MB, ng/ml | 7.70 (1.21, 5.56) | 7.90 (1.15, 5.67) | 7.44 (1.20, 5.07) | 0.834 | |
| Myoglobin, ng/ml | 95.75 (43.80, 109.47) | 95.97 (44, 109) | 95.47 (38, 114) | 0.785 | |
| cTnT, ng/ml | 0.60 (0.22, 0.58) | 0.41 (0.22, 0.58) | 0.85 (0.21, 0.65) | 0.955 | |
| NT, proBNP, pg/l | 3481 (409, 3554) | 2559 (194, 3572) | 4644 (962, 3820) | 0.240 | |
| Blood glucose, mmol/l | 4.84 (4.06, 5.06) | 4.60 (4.13, 5.05) | 5.15 (3.90, 5.07) | 0.994 | |
| Glycated hemoglobin, % | 6.06 (5.51, 6.20) | 6.24 (5.52, 6.20) | 5.82 (5.42, 6.11) | 0.229 | |

Data are mean ± SD or median (P_25_, P_75_), the categorical variables are presented as absolute numbers (percentages). Group differences were assessed by chi-square test or Kruskal-Wallis H tests. ALT = alanine aminotransferase; AST = aspartate aminotransferase; BE = base excess; BUN = Blood urea nitrogen; CREA = creatinine; HCO_3_^-^ = bicarbonate concentration; HGB = hemoglobin; NYHC-FC = New York Heart Association-functional class; PaCO_2_ = carbon dioxide partial pressure; PaO_2_ = oxygen partial pressure; PT = prothrombin time; RBC = erythrocyte; TC = total cholesterol; TG = triglyceride; UA = uric acid; BNP = B-Type natriuretic peptides; CRP = C-reactive protein; FIB = fibrinogen; HDL-C = high-density lipoprotein; LDL-C = low-density lipoprotein; Neu% = neutrophilic granulocyte percentage; PaCO_2_ = carbon dioxide partial pressure; PaO_2_ = oxygen partial pressure; DBP = diastolic blood pressure; SBP = systolic blood pressure. ^a^*P* <0.05: The group difference assessed by the Two-tailed unpaired t test or Mann-Whitney U test was significant.

**Supplementary Table 2 Comparisons of CTA parameters**

| **Characteristic** | **Total** | **PE** | **CTEPH** | ***P* value** |
| --- | --- | --- | --- | --- |
| Left lower PAD, mm | 12.5 (10.3, 14.1) | 12.6 (10.4, 14.2) | 12.3 (10.0, 14.2) | 0.381 |
| Left lower PBD, mm | 8.6 (7.0, 9.6) | 8.3 (6.9, 9.5) | 8.9 (7.0, 10.7) | 0.778 |
| Left upper PAD, mm | 17.7 ± 5.0 | 17.9 ± 4.9 | 17.3 ± 5.1 | 0.774 |
| Left upper PBD, mm | 11.4 ± 2.3 | 11.4 ± 1.9 | 11.4 ± 5.1 | 0.002^a^ |
| Right intermediate PAD, mm | 16.7 ± 3.5 | 17.1 ± 3.4 | 16.3 ± 3.6 | 0.755 |
| Right intermediate PBD, mm | 11.3 ± 5.0 | 11.1 ± 2.0 | 11.6 ± 2.4 | 0.249 |
| Right upper PAD, mm | 14.5 (11.9, 15.9) | 14.3 (11.9, 15.9) | 14.6 (11.9, 17.1) | 0.962 |
| Right upper PBD, mm | 12.5 (10.9, 14.5) | 12.7 (11.1, 14.2) | 12.2 (10.3, 14.7) | 0.334 |
| rSIVC | 1.2 ± 0.2 | 1.2 ± 0.2 | 1.1 ± 0.2 | 0.488 |
| Left rPA | 0.7 (0.6, 0.7) | 0.7 (0.6, 0.7) | 0.7 (0.6, 0.7) | 0.936 |
| Right rPA | 0.7 (0.6, 0.8) | 0.7 (0.6, 0.8) | 0.7 (0.6, 0.8) | 0.288 |

Data are mean ± SD or median (P_25_, P_75_). PAD = pulmonary artery diameter; PBD = pulmonary bronchus diameter; rSIVC = the ratio of superior to inferior vena cava diameter; rPA = the ratio of pulmonary artery to ascending aorta diameter. ^a^*P* <0.05: The group difference assessed by the Two-tailed unpaired t test or Mann-Whitney U test was significant.
